# Pharmacovigilance: A Worldwide Master Key for Drug Safety Monitoring

**DOI:** 10.4103/0975-1483.66802

**Published:** 2010

**Authors:** G Jeetu, G Anusha

**Affiliations:** *Department of Pharmacy Practice, Manipal College of Pharmaceutical Sciences, Manipal, Karnataka, India*; 1 *Institute of Professional Studies College of Pharmacy, Gwalior, Madhya Pradesh, India*

**Keywords:** Drug safety, erice declaration, pharmacovigilance

## Abstract

Pharmacovigilance is like a sunshade to describe the processes for monitoring and evaluating ADRs and it is a key component of effective drug regulation systems, clinical practice and public health programmes. The number of Adverse Drug Reactions (ADRs) reported resulted in an increase in the volume of data handled, and to understand the pharmacovigilance, a high level of expertise is required to rapidly detect drug risks as well as to defend the product against an inappropriate removal. The current global network of pharmacovigilance centers, coordinated by the Uppsala Monitoring Centre, would be strengthened by an independent system of review. This would consider litigious and important drug safety issues that have the potential to affect public health adversely beyond national boundaries. Recently, pharmacovigilance has been confined, mainly to detect adverse drug events that were previously either unknown or poorly understood. Pharmacovigilance is an important and integral part of clinical research and these days it is growing in many countries. Today many pharmacovigilance centers are working for drug safety monitoring in this global pitch, however, at the turn of the millennium pharmacovigilance faces major challenges in aspect of better safety and monitoring of drugs. In this review we will discuss about drug safety, worldwide pharmacovigilance centers and their role, benefits and challenges of pharmacovigilance and its future consideration in healthcare sectors.

## INTRODUCTION

Drug safety and pharmacovigilance remains a dynamic clinical and scientific discipline. Pharmacovigilance is defined by the World Health Organization (WHO) as ’the science and activities relating to the detection, assessment, understanding and prevention of adverse effects or any other drug-related problem’;[1] it plays a vital role in ensuring that doctors, together with the patient, have enough information to make a decision when it comes to choosing a drug for treatment.[2] However, despite all their benefits, evidence continues to get those bigger adverse reactions to medicines which are common, yet often preventable, cause of illness, disability and even death. In some countries, adverse drug reactions (ADRs) rank among the top 10 leading causes of mortality. In order to prevent or to reduce harm to patients and thus improve public health, mechanisms for evaluating and monitoring the safety of medicines in clinical use are vital.[1] Pharmacovigilance programs in the next 10 years, describe in brief the potential implications of such trends on the evolution of the science. These days pharmacovigilance is facing lots of challenges to develop better health care systems in this global pitch. Major challenges are globalization, web-based sales and information, broader safety concerns, public health versus pharmaceutical industry economic growth, monitoring of established products, developing and emerging countries, attitudes and perceptions to benefit and harm, outcomes and impact.[3]

## HISTORICAL PERSPECTIVES OF WHO - DRUG SAFETY MONITORING

In 2002, more than 65 countries have their own pharmacovigilance centers. Membership of the WHO for International Drug Monitoring is coordinated by the WHO Collaborating Centre for International Drug Monitoring, known as the Uppsala Monitoring Centre (UMC). Pharmacovigilance is now firmly based on sound scientific principles and is integral to effective clinical practice. The discipline needs to develop further to meet public expectations and the demands of modern public health. The Sixteenth World Health Assembly adopted a resolution (WHA 16.36)[5] that reaffirmed the need for early action in regard to rapid dissemination of information on adverse drug reactions and led later to creation of the WHO Pilot Research Project for International Drug Monitoring. The purpose of this was to develop a system, applicable internationally, for detecting previously unknown or poorly understood adverse effects of medicines.[6]

## WORLDWIDE SOLDIERS OF PHARMACOVIGILANCE

A complex and vital relationship exists between wide ranges of partners in the practice of drug safety monitoring. These partners must jointly anticipate, understand and respond to the continually increasing demands and expectations of the public, health administrators, policy officials, politicians and health professionals.

*The Quality Assurance and Safety:* The team is a part of the Department of Essential Drugs and Medicines Policy, within the WHO Health Technology and Pharmaceuticals cluster. The purpose of the department is to help save lives and improve health by closing the huge gap between the potential that essential drugs have to offer and the reality that for millions of people, particularly the poor and disadvantaged, medicines are unavailable, unaffordable, unsafe or improperly used.[7]

*The Uppsala Monitoring Centre:* The principal function of the Uppsala Monitoring Centre is to manage the international database of ADR reports received from National Centers.[8] The UMC has established standardized reporting by all National Centers and has facilitated communication between countries to promote rapid identification of signals.

*The National Pharmacovigilance Centers:* National Centers have played a significant role in increasing public awareness of drug safety. This development is partly attributable to the fact that many national and regional centers are housed within hospitals, medical schools or poison and drug information centers, rather than within the confines of a drug regulatory authority. Major centers in developed countries have established active surveillance programmes using record linkage and prescription event monitoring systems (PEM) to collect epidemiological information on adverse reactions to specific drugs. Such systems have already been implemented in New Zealand, the United Kingdom, Sweden and the United States of America. The entire cost of a pharmacovigilance system, compared with the national expenditure on medicines or the cost of ADRs to the nation is very small indeed.[9,10]

*Hospitals and Academia:* A number of medical institutions have developed adverse reaction and medication error close watch systems in their clinics, wards and emergency rooms. Case-control studies and other pharmacoepidemiological methods have increasingly been used to estimate the harm associated with medicines once they have been marketed. Academic centers of pharmacology and pharmacy have played an important role through teaching, training, research, policy development, clinical research, ethics committees (institutional review boards) and the clinical services they provide.[11–13]

*Health Professionals:* Originally physicians were the only professionals invited to report as judging whether disease or medicine causes a certain symptom by exercising the skill of differential diagnosis. Today, different categories of health professionals will observe different kinds of drug related problems.[14,15]

*Patients:* Only a patient knows the actual benefit and harm of a medicine taken. Direct patient participation in the reporting of drug related problems will increase the efficiency of the pharmacovigilance system and compensate for some of the shortcomings of systems based on reports from health professionals only.

## PHARMACOVIGILANCE IN DRUG REGULATION

Pharmacovigilance programs made strong by links with regulators. Regulators understand that pharmacovigilance plays a specialized and pivotal role in ensuring ongoing safety of medicinal products.

*Clinical trial regulation:* In recent years there has been a substantial increase in the number of clinical trials in developed and developing countries. In their approval of clinical trials, regulatory bodies look at safety and efficacy of new products under investigation. Safety monitoring of medicines in common use should be an integral part of clinical practice. Education and training of health professionals in medicine safety, exchange of information between national pharmacovigilance centers, the coordination of such exchange, and the linking of clinical experience of medicine safety with research and health policy, all serve to enhance effective patient care. A regular flow and exchange of information in this way means that national pharmacovigilance programmes are ideally placed to identify gaps in our understanding of medicine-induced diseases.[1]

*Post marketing safety drug monitoring:* These includes detection of drug interactions, measuring the environmental burden of medicines used in large populations, assessing the contribution of ’inactive’ ingredients to the safety profile, systems for comparing safety profiles of similar medicines, surveillance of the adverse effects on human health of drug residues in animals, e.g. antibiotics and hormones. The Council for International Organizations of Medical Sciences (CIOMS) report on benefit-risk assessment of medicines after marketing has contributed to a more systematic approach to determining the merit of available medicines.[16]

*Pharmacovigilance in national drug Policy:* The provision of good quality, safe and effective medicines and their appropriate use is the responsibility of national governments. Multidisciplinary collaboration is of great importance in particular, links need to be forged between various departments of the ministry of health and also with other stakeholders, such as the pharmaceutical industry, universities, nongovernmental organizations (NGOs) and those professional associations having responsibility for education on rational use of medicines and pharmacotherapy monitoring.

*Pharmacovigilance in Disease Control Public Health Programmes:* The monitoring of medicine safety in countries where there is no regulatory or safety monitoring system in place, or in remote areas with little or no health care surveillance or infrastructure, has been identified as a matter for concern. The problems are especially apparent in situations that involve the use of medicines in specific communities, for example, for the treatment of tropical diseases such as malaria, leishmaniasis and schistosomiasis, and for the treatment of HIV/AIDS and tuberculosis. Pharmacovigilance should be a priority for every country with a public health disease control programs.[1]

## PHARMACOVIGILANCE AND INTERNATIONAL HEALTH

The current global network of pharmacovigilance centers is coordinated by the Uppsala Monitoring Centre, would be strengthened by an independent system of review. This would consider contentious and important drug safety issues that have the potential to affect public health adversely beyond national boundaries. The Erice Declaration provides a framework of values and practice for collection, analysis and subsequent communication of drug safety issues. Today, the burden of ADRs on public health despite the progress in pharmacovigilance that has been made, the burden on public health of ADRs remains significant.[17] Pharmacoeconomic studies on the costs of adverse reactions suggest that governments pay considerable amounts from health budgets towards covering costs associated with them.[18] However, it has become increasingly clear that the safety profile of medicines is directly linked with socio-political, economic and cultural factors that in turn affect access to medicines, their utilization patterns and public perceptions of them.[19,20]

*Drug utilization:* Drug utilization patterns are a major determinant in drug safety. For instance, the use of injectable medicines is more common in developing countries.[21] Direct advertizing to the consumer of prescription medicines has become commonplace in many countries. With this information patients feel more able to make their own therapeutic decisions, without assistance from doctor or pharmacist. The result has been increasing self medication, licit and illicit sale of medicines over the Internet, and over-prescribing by doctors on patients’ demand. This has had considerable effect on increased prescribing.[22,23] Such public health programmes, however, need not focus only on patients but could be used for the benefit of the general public as well. Such awareness-building and educational initiatives should also include children and elderly populations and could be greatly facilitated through partnerships with the media, educational institutions, governmental and non-governmental organizations. The success of WHO International Drug Monitoring Programmes is entirely dependent on the contributions of national pharmacovigilance centers. Ideally every country should have a pharmacovigilance centre.[1]

## THE ERICE DECLARATION

The Erice Declaration represented significant progress in the light of these changes for pharmacovigilance The Declaration challenges all the players like public health administration, health professionals, the pharmaceutical industry, government, drug regulators, the media, consumers to strive towards the highest ethical, professional and scientific standards in protecting and promoting safe use of medicines. The Declaration urges governments and others involved in determining policies relating to the benefit, harm, effectiveness and risk of medicines to account for what they communicate to the public and patients.

*Challenges for the Erice Declaration:* There are several challenges facing pharmacovigilance programmes in achieving the aspirations of the Erice Declaration. Like The difficulties and risks in communicating conflicting or contentious messages to the public. For instance, during the course of immunization programmes, communication of new safety concerns associated with the vaccine(s) or with programmatic errors may result in a dramatic fall in coverage. Nonetheless, an approach of secrecy in such circumstances is likely to erode public trust and confidence, and it fails to respect the rights of the public to participate in decision-making. Not only do facts and figures need to be shared with the public, but also the process by which the data is assessed and how decisions are made should be shared openly. Another challenge is Communication between national drug regulatory authorities and national pharmacovigilance centers needs to be improved so that regulatory decisions with possible international implications are rapidly communicated to regulators, to avoid widespread public concern or panic.[24]

## INTERNATIONAL RESPONSE TO DRUG SAFETY ISSUES

Certain safety issues are likely to have a global impact with possibly serious consequences for public health. When this happens, a cohesive international assessment and response is needed. The WHO has supported the creation of an independent advisory panel composed of a broad spectrum of medical disciplines including clinical pharmacologists, regulators, academics and epidemiologists. The functions of this panel will be to provide advice to WHO on safety issues relating to medicinal products, including its Collaborating Centre for International Drug Monitoring and through it to the Member States of WHO.[25]

## NEWS BROADCAST RELATED TO PANDEMIC PHARMACOVIGILANCE UPDATE

The benefit and risk balance of the pandemic vaccines and antiviral used for the current H1N1 influenza pandemic continues to be positive. To date, no unexpected serious safety issues have been identified. The most frequent adverse reactions that have been reported are non-serious and as expected. The EMEA issued a press release on November 2009 reaffirming the efficacy and safety of the centrally authorized vaccines. With vaccination campaigns ongoing in the European Union, it is estimated that about 10 million people have been vaccinated so far. The vaccine adverse effects reported so far have mainly been symptoms such as fever, nausea, headache, allergic reactions and injection site reactions, confirming the expected safety profile of the three vaccines. New clinical trial data showed greater incidence of fever following the second dose of Pandemrix in infants from 6 months to 35 months. An assessment of these data is ongoing.[26]

## CONSIDERATIONS FOR THE FUTURE AND ITS CHALLENGES

Some of the serious challenges facing pharmacovigilance programmes in the next ten years, describing in brief the potential implications of such trends on the evolution of the science.

Some key points for future consideration which may be improved to make better pharmacovigilance practice:

1. Pharmacovigilance should be less focused on finding harm and more on extending knowledge of safety.
2. Complex risk-benefit decisions are amenable to, and likely to be improved by, the use of formal decision analysis.
3. Pharmacovigilance should operate in a culture of scientific development. This requires the right balance of inputs from various disciplines, a stronger academic base, and greater availability of basic training, and resource which is dedicated to scientific strategy.
4. Systematic audit of pharmacovigilance processes and outcomes should be developed and implemented based on agreed standards (’good pharmacovigilance practice’).

Some Major challenges face pharmacovigilance are as follows:

*Globalization:* The globalization of drug distribution and the increased exposure of massive populations to large volumes of medicines. These include novel chemical entities used for symptomatic relief and lifestyle modification as well as medicines used in developing countries to curb the prevalence of pandemic diseases such as HIV/AIDS, malaria and tuberculosis.

*Web-based sales and information:* The Internet, in addition to its many benefits, has also facilitated the uncontrolled sale of medicines across national borders. Drug information in all forms and with varying levels of accuracy is distributed internationally through this medium. Such information covers prescription drugs, unregistered medicines, highly controlled substances and traditional and herbal medicines with questionable safety, efficacy and quality.

*Broader safety concerns:* The scope of pharmacovigilance continues to broaden as the array of medicinal products grows. There is a realization that drug safety is more than the monitoring, detection and assessment of ADRs occurring under clearly defined conditions and within a specific dose range. Rather, it is closely linked to the patterns of drug use within society. Problems resulting from irrational drug use, overdoses, polypharmacy and interactions, increasing use of traditional and herbal medicines with other medicines, illegal sale of medicines and drugs of abuse over the Internet increasing self medication practices substandard medicines, medication errors, lack of efficacy are all within the domain of pharmacovigilance. Current systems need to evolve in order to address this broad scope adequately.

*Public health versus pharmaceutical industry economic growth:* There may be shortcomings and at times conflicting interests within the pharmaceutical industry when dealing with public health concerns arising from drug safety issues. The industry needs to overcome weaknesses in safety monitoring during clinical trials and post-marketing surveillance.

*Monitoring of established products:* The generic sector of the pharmaceutical industry has not fully recognized and its responsibility to continuously monitor the safety of its products throughout the world. There is the erroneous belief that generic drugs are inherently safe even when they interact with other medicines. The generic sector is the largest supplier of essential drugs.

*Attitudes and perceptions to benefit and harm:* These trends have dramatically changed the way in which medicines are used by society. Healthcare providers, patients and the public have responded in different ways to these changing trends as has been described in previous chapters. Their perception of benefit and harm and the level of acceptable risk for medicines in the face of these rapid developments have not been considered in a meaningful way. The harm caused by medicines has been shown to be significant. Morbidity and mortality from drug-induced diseases are only recently being recognized as an important item on the public health agenda in developed and developing countries.

*Outcomes and Impact:* Along with increased public awareness over safety of medicines, there is an increasing public stare on the performance of the health professions, industry and regulators. Increased accountability must lead to more research into the effectiveness of pharmacovigilance and its place in improving public perception. A major focus must be to empower health practitioners and patients themselves with useful information that improves individual therapy, aids the diagnosis and management of medicine-induced disease, and generally leads to a reduction of iatrogenic diseases.[27–29]

## CONCLUSION

Pharmacovigilance continues to play a crucial role in meeting the challenges posed by the ever increasing range and potency of medicines, all of which carry an inevitable and some- times unpredictable potential for harm. When adverse effects and toxicity do appear, especially when previously unknown, it is essential that these are reported, analyzed and their significance is communicated effectively to the audience having knowledge to interpret the information. For all medicines, there is a trade-off between the benefits and the potential for harm. The harm can be minimized by ensuring that medicines of good quality, safety and efficacy are used rationally, and that the expectations and concerns of the patient are taken into account when therapeutic decisions are made. To achieve this is to serve public health, and to foster a sense of trust among patients in the medicines they use that would extend the confidence in the health service in general, ensure that risks in drug use are anticipated and managed, provide regulators with the necessary information to amend the recommendations on the use of the medicines, improve communication between the health professionals and the public and educate health professionals to understand the effectiveness or risk of medicines that they prescribe.
